# Evaluation of HER2 and Nectin-4 Expression in Primary Vaginal Cancers

**DOI:** 10.3390/ijms27157018

**Published:** 2026-08-05

**Authors:** Gilbert Georg Klamminger, Hanna Wattendorff, Felix Hässlin, Christoph Eckert, Silke Mitschke, Stefanie Zimmer, Meletios P. Nigdelis, Roxana Schwab, Walburgis Brenner, Annette Hasenburg, Yaman Degirmenci

**Affiliations:** 1 Department of Obstetrics and Gynecology, University Medical Center of the Johannes Gutenberg University Mainz, 55131 Mainz, Germany; 2 Institute of Pathology, University Medical Center of the Johannes Gutenberg University Mainz, 55131 Mainz, Germany; 3 TRON—Translational Oncology Mainz gGmbH, 55131 Mainz, Germany; 4 Tissue Bank of the University Medical Center Mainz, 55131 Mainz, Germany; 5 Department of Gynecology, Obstetrics and Reproductive Medicine, Saarland University Medical Center (UKS), 66424 Homburg, Germany

**Keywords:** primary vaginal cancer, druggable targets, tumor-agnostic therapy, HER2, Nectin-4

## Abstract

This study assessed the prevalence and clinical relevance of p16^INK4a^, p53, HER2, and Nectin-4 protein expression in primary vaginal cancer (PVC), with HER2 and Nectin-4 representing potentially druggable targets. A total of 34 patients with PVC from the University Medical Center Mainz biobank were included. Immunohistochemistry was used to evaluate the expression of p16, p53, HER2, and Nectin-4: p16, p53, and HER2 staining results were dichotomized (positive vs. negative; mutant vs. wildtype staining pattern), whereas Nectin-4 expression was assessed using an immunoreactive score (IRS) based on staining intensity and the proportion of positive tumor cells. Univariate analyses were performed to identify intergroup differences, and survival analyses were conducted using the log-rank test. Negative p16 expression was significantly associated with adenocarcinomatous histology (*p* = 0.0211), but neither p16 nor p53 status showed prognostic relevance. HER2 positivity was detected in 14.7% of tumors and was significantly associated with adenocarcinoma (*p* = 0.0001), p16 negativity (*p* = 0.0035), and younger age (*p* = 0.0483). Based on IRS, strong total Nectin-4 expression was observed in 20.59% and moderate membranous Nectin-4 expression in 17.65% of cases. No significant associations were found between HER2 or Nectin-4 expression and clinical outcomes; however, a limited statistical power of the survival analysis due to the low number of events (n = 10 deaths) should be considered. This analysis demonstrates a notable prevalence of HER2 and Nectin-4 expression in PVC.

## 1. Introduction

Despite recent advances in diagnostics - such as classifications increasingly grounded in tumorigenesis (e.g., the association with HPV infection) - the rarity of primary vaginal cancer (PVC, accounting for 1–2% of all female genital tract malignancies worldwide) has limited the available body of research [1,2,3]. Accordingly, international guidelines and recommendations, including those issued by the National Comprehensive Cancer Network (NCCN) and the European Society of Gynaecological Oncology (ESGO), frequently depend on extrapolating diagnostic and therapeutic recommendations from evidence derived from vulvar and cervical cancers [4,5].

Since the era of precision medicine has brought tumor-agnostic therapies and individualized treatment strategies to the forefront, research has increasingly focused on the identification of actionable molecular and protein targets. In patients with advanced/progressive, recurrent, or metastatic PVC, the current NCCN guidelines recommend not only PD-L1 (programmed death-ligand 1) and HER2 (Human Epidermal Growth Factor Receptor 2) assessment by immunohistochemistry (IHC), but also consideration of additional molecular testing to identify potential tumor-agnostic therapeutic targets, including microsatellite instability (MSI), tumor mutational burden (TMB), NTRK or RET fusions [5]. However, only a limited number of studies to date have evaluated and reported on the identification of potentially targetable molecular alterations in this specific entity. Also in this context, the majority of available evidence is derived from studies conducted in vulvar cancer, in which the expression of potentially targetable biomarkers—such as HER2, hormone receptors, TROP2 (Trophoblast Cell Surface Antigen 2), Nectin-4 (poliovirus receptor-like 4), FOLR1 (Folate Receptor Alpha), CLDN18.2 (Claudin-18.2), and PD-L1—has been systematically examined [6,7,8]. Against this background, we assembled a cohort of PVC and conducted a monocentric, retrospective clinicopathological study aimed at (a) characterizing clinicopathological features with respect to IHC expression of p16 (HPV-association) and p53 (mutation/wildtype staining patterns), and (b) assessing the prevalence and prognostic significance of immunohistochemical expression of the potentially druggable targets HER2 and Nectin-4, for both of which specific antibody-drug conjugates (ADC) (HER2: trastuzumab deruxtecan (T-Dxd); Nectin-4: enfortumab vedotin (EV)) are currently approved for cancer treatment [9,10]. Among the potential biomarkers listed above, HER2 and Nectin-4 were prioritized given the availability of approved antibody–drug conjugates, evidence of biological activity in other epithelial tumors [9,11,12], and - especially for HER2 - well-established, standardized immunohistochemical assays.

## 2. Results

### 2.1. Clinical Data

**Figure 1:**
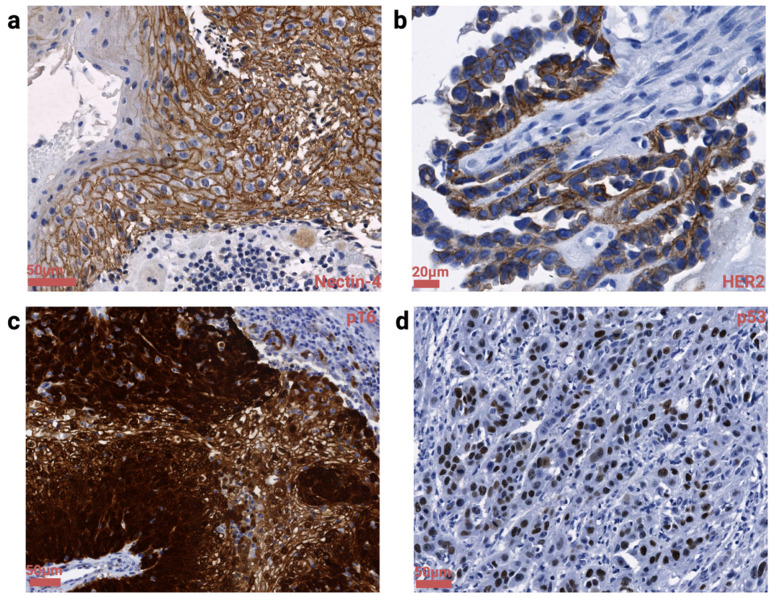
Visualization of biomarker-specific staining patterns in our cohort of PVC. (**a**) Nectin-4 expression with a distinct and predominant membranous aspect (IRS_total_: strong, IRS_memb_: moderate). (**b**) Display detail of an adenocarcinoma with positive HER2 expression (IHC:2+). (**c**) p16 positivity (so-called abnormal diffuse positive staining pattern). (**d**) Vaginal squamous cell carcinoma (SCC) with parabasal/diffuse overexpression of p53 indicating a mutant staining pattern. Created in BioRender Klamminger G.G. (2026) https://BioRender.com/b3yevq1.

Altogether, 34 patients with PVC and sufficient tissue for biomarker analysis were included in this study. Surgical specimens were available in 19 cases (55.88%), while biopsy material was analyzed in 15 cases (44.12%). Histologically, 30 patients (88.24%) were diagnosed with squamous cell carcinoma (SCC) and 4 (11.76%) with adenocarcinoma NOS (not otherwise specified). The median age of the cohort was 62 years (IQR: 53–73 years). At initial diagnosis, 11 patients (32.35%) presented with FIGO stage I, 6 (17.65%) with stage II, 11 (32.35%) with stage III, 2 (5.88%) with stage IVA, and 4 (11.76%) with stage IVB disease. Regarding first-line treatment, 13 patients (38.24%) underwent surgery alone, 12 (35.29%) received chemoradiotherapy, and 9 (26.47%) were treated with surgery followed by radiotherapy and/or chemoradiotherapy. During follow-up, 14 patients (41.18%) developed local recurrence and 12 (35.29%) developed distant metastases. At the end of the study period, 10 patients (29.41%) had died after a median follow-up time of 24 (IQR: 9.8–44.8) months. The clinical information is listed in Table 1, and an overview of different IHC staining patterns is presented in Figure 1. The initial Shapiro–Wilk test indicated a non-normal distribution for all variables of interest, except patient age (*p* = 0.5884).

### 2.2. IHC: p16 and p53 Staining Patterns

Among the study cohort, 27 (79.41%) lesions were p16 positive (Figure 1c), while 7 (20.59%) cases were p16 negative. A total of 5 (14.71%) cases were classified with a p53 mutant staining pattern, while 29 (85.29%) cases showed a p53 wildtype staining pattern. Individual staining patterns of SCC are listed in Table S1; within p53 mutant cases of SCC, solely a basal overexpression or parabasal/diffuse overexpression (Figure 1d) could be observed, while absent or cytoplasmic detection of p53 was not identified. Negative p16 staining was significantly associated with adenocarcinomatous histology (***p*** **= 0.0211**, OR = 19.50, 95% CI = 2.116–258.6; Fisher’s exact test), while p53 showed no association with histological type (*p* = 0.4879, Fisher’s exact test). Double-positivity (positivity for p16 plus p53 mutant staining pattern) was observed in one case. Within our cohort, neither expression of p16 (recurrence: *p* = 0.4099, metastasis: *p* > 0.9999, both Fisher’s exact test; overall survival (OS): *p* = 0.1232, HR = 2.546, 95% CI: 0.55–11.74; log-rank test) nor p53 (recurrence: *p* = 0.6272, metastasis: *p* = 0.6347, both Fisher’s exact test; OS: *p* = 0.3428, log-rank test) yielded significant prognostic relevance (refer also to Tables S2 and S3 for details).

### 2.3. IHC: HER2 and Nectin-2 Staining Patterns

In the present study population, 29 (85.29%) neoplasms were classified as HER2 negative (HER2 score 0 or 1), while 5 (14.7%) were classified as HER2 positive (HER2 score 2 or 3) (refer also to Table 2 and Figure 1b). All four adenocarcinomas were classified as HER2 positive. HER2 positivity was significantly associated with adenocarcinomas (*p* = 0.0001, OR not reliably estimable due to complete separation; Fisher’s exact test), as well as negative p16 expression (*p* = 0.0035, OR = 0.02885, 95%CI = 0.00232–0.32; Fisher’s exact test) and younger patients (median age 48 years; *p* = 0.0483, Welch’s *t*-test) (see Table 3). HER2 did not show a significant relevance with regard to recurrence (*p* > 0.9999, Fisher’s exact test), metastasis (*p* = 0.6347, Fisher’s exact test) or OS (*p* = 0.4905, log-rank test).

The detailed prevalence of Nectin-4 staining expression is listed in detail in Table 2. According to the IRS_total_ score, 8 (23.53%) patients showed moderate staining and 7 (20.59%) showed strong staining; with regard to the IRS_memb_ score, 6 (17.65%) patients showed moderate staining (Figure 1a) and 7 (20.59%) showed weak protein expression. Nectin-4 expression was not associated with p16 status (IRS_total_: *p* = 0.0623; IRS_memb_: *p* = 0.6387, both by Mann–Whitney test) or histological type (IRS_total_: *p* = 0.1595; IRS_memb_: *p* = 0.5427, both by Mann–Whitney test). Evaluating the optimal cut-off value for survival analysis with regard to Nectin-4 expression, the highest Youden’s index (YI) was identified for IRS_total_ < 1.5 (YI = 0.2) and IRS_memb_ < 0.5 (YI = 0.1167). Within our cohort, Nectin-4 expression did not yield prognostic significance in terms of recurrence, metastasis or survival, neither stratified by IRS_total_ (recurrence: *p* = 0.1509, metastasis: *p* = 0.8407, both Mann–Whitney test; OS: *p* = 0.4635, log-rank test) nor IRS_memb_ (recurrence: *p* = 0.2698, metastasis: *p* = 0.3872, both Mann–Whitney test; OS: *p* = 0.6705, log-rank test). Table S4 provides a tabular overview. To assess potential sampling bias related to tissue type (surgical resection specimens versus biopsy specimens), we evaluated whether biomarker expression differed according to the source of tissue material and did not determine a corresponding statistically significant spatial antigen density in biomarker expression (HER2: *p* > 0.9999, Fisher’s exact; IRS_memb_: *p* = 0.3418 and IRS_total_: *p* = 0.1999, both Mann–Whitney test).

## 3. Discussion

### 3.1. Summary of Main Results

In this retrospective analysis of PVC, 79.41% of tumors demonstrated p16 positivity, thereby meeting the diagnostic criteria for HPV-associated carcinoma, whereas 14.71% exhibited an aberrant p53 immunohistochemical pattern. No significant associations were observed between p16 or p53 status and recurrence, metastatic spread, or OS. Importantly, 14.7% of tumors were identified as HER2-positive (IHC score 2+/3+), indicating potential eligibility for tumor-agnostic treatment with the ADC T-Dxd. That said, we showed that patients with HER2 positivity were younger and more frequently had adenocarcinomas and an HPV-independent tumorigenesis. Furthermore, our results confirmed a high prevalence of Nectin-4 expression (IRS_total_), with approximately 82% of cases exhibiting positive protein expression across a range of staining intensities, while weak to moderate membranous Nectin-4 expression (IRS_memb_) was observed in approximately 38% of cases. To our knowledge, this represents the first description of Nectin-4 expression in PVC. Collectively, these exploratory and hypothesis-generating findings provide valuable preliminary insights into biomarker landscapes in PVC but require validation in larger, prospective cohorts prior to translation into clinical practice.

### 3.2. Results in the Context of Published Literature

Since current diagnostic and therapeutic evidence is largely derived from vulvar cancer studies rather than vaginal cancer, only a few studies evaluated the impact of p16 and p53 staining in PVC. A meta-analysis from 2019 described a 66.7% (95% CI = 54.7–77.8) prevalence of HPV association in vaginal SCC and a prevalence of 85.2% (95% CI = 78.2–91.0) in its precursors. Positivity for p16 was identified in 89.9% (95% CI = 81.7–94.6) of vaginal SCC. Although p16 positivity was described in 38.9% (95% CI = 0.9–90.0) of HPV-independent SCC, the *2020 WHO Classification of Female Genital Tumors* lists p16 evaluation as an essential diagnostic criterion to determine HPV association, while a molecular workup of HPV-DNA is not explicitly mandatory [13]. As outlined by a meta-analysis from Rasmussen et al. (2021), most studies determined a positive association of p16 positivity and improved OS in PVC [14]; a finding also in line with a subsequent, more recent study [15]. Despite a visually apparent separation of the Kaplan–Meier curves (Figure S1), no significant association between p16 expression and OS was identified in our cohort. This lack of significance may be attributable to the limited number of events (n = 10), despite a cohort size consistent with biomarker studies in PVC. However, the wide confidence intervals observed indicate potential limited precision and suggest that increased statistical power, either through extended follow-up or larger sample size, may alter our observed results.

Interestingly, we determined only one lesion to be double-positive (IHC positivity for p16 and p53 mutant staining pattern)—while a recent study by Egger et al. of 22 cases with PVC identified six double-positive cases among nine cases with mutant p53 staining pattern [15]. Double-positivity occurs between 0 and 6.8% in vulvar cancer, although its clinical significance has not yet been established [16]. It is known that HPV-associated cases could also exhibit aberrant p53 staining patterns, most likely due to viral E6 oncoprotein degradation of p53 [14,17,18]; indeed, a secondary dysregulation of p16^INK4a^, as in high-grade serous ovarian cancer, could also be imaginable. Current expert opinions in vulvar cancer suggest that tumors with double-positivity are most likely HPV-independent p53 mutant vulvar SCC [16]. Given the scope and exploratory set-up of this study, analyses were restricted to a descriptive level, and the question of the underlying biological nature of double-positive cases is deferred to future studies. We therefore advocate for consistent p53 testing even in p16-positive cases [19], which could be readily integrated into routine clinical workflows. As presented in our analysis, most studies do not report a significant association of p53 status and OS in PVC [14,15].

Targeting the transmembrane tyrosine kinase receptor HER2 (member of the EGFR family), T-Dxd consists of an immunoglobulin G1 anti-HER2 monoclonal antibody conjugated to a topoisomerase I inhibitor [20]. Contributing to cell proliferation and differentiation via the phosphoinositide 3-kinase (PI3K)/Akt pathway and the mitogen-activated protein kinase (MAPK) cascade [21], HER2 overexpression represents a common molecular alteration across diverse neoplasms, especially in malignancies of epithelial origin [22]; it is linked to tumor aggressiveness as well as adverse clinical outcomes [23,24,25]. In line with an accelerated approval (based on several phase II trials) by the U.S. Food and Drug Administration (FDA) in 2024, which enables T-Dxd application in adults with unresectable or metastatic HER2-IHC 3+ solid tumors after prior systemic treatment [9,26,27,28], the current NCCN guideline advocates for the assessment of tumor-agnostic therapeutic targets, including HER2 status, in PVC [5]. Within our study, we defined HER2 positivity according to the DESTINY-PanTumor02 trial criteria, where IHC scores of 3+ or 2+ were considered indicative of HER2 overexpression in the so-called ‘other tumor cohort’—which, among others, included one vulvar carcinoma [9]. Our presented work shows that approximately 15% of PVCs indeed are HER2 positive, particularly among adenocarcinomas and p16-negative tumors, highlighting a potential role for tumor-agnostic therapy with T-Dxd. While expression of HER2 in female patients with extramammary Paget disease is estimated to be approx. 32% (95% CI = 0.27–0.38) [29], it has been reported in only around 0.9–1.9% of vulvar SCC [30,31]. Evidence within PVC remains sparse [32,33], as also highlighted in a recent review by Zannoni et al. [21]. Our reported prevalence rate is in line with an examination by Skomedal et al., who immunohistochemically evaluated 46 cases of PVC and determined a positive HER2 expression in 11% of cases—interestingly in four SCC and only in one adenocarcinoma [34]. That said, we hypothesize a biological similarity between HER2-expressing adenocarcinomas of the vagina and the cervix uteri, where rates of 4.1% in SCC and 10.3% in non-SCC have been reported [35]. Nevertheless, extensive molecular profiling is necessary to establish whether PVCs are truly most accurately aligned with cervical cancers also at the molecular level [36]. To date, the distinct role of HPV-association in HER2-positive carcinomas of the lower female genital tract remains controversial, with studies reporting HPV-dependent upregulation of HER2 and vice versa [21].

The transmembrane receptor Nectin-4 is a cellular adhesion molecule (CAM) involved in cancer cell migration and proliferation and generally expressed in around 69% of solid tumors [11,12,37]. The ADC EV (human IgG1 kappa monoclonal nectin-4 antibody plus the cytotoxic microtubule targeting agent monomethyl auristatin E) is currently FDA approved for urothelial cancer treatment [11,38]. Contrasting ongoing trials targeting Nectin-4 in cervical cancer [12,39] or penile cancer [40], the evidence of Nectin-4 expression and its clinical relevance in vulvar cancer and PVC remains limited. In vulvar cancer, moderate to strong Nectin-4 expression is reported in 32.4% to 92.7% of cases; however, this varies depending on the mode of staining interpretation (IRS vs. H-score). Nectin-4 expression has been reported to be associated with HPV-related vulvar tumors but does not appear to be significantly associated with improved survival [41,42]. No distinct Nectin-4 amplification could be detected in vulvar cancer using fluorescence in situ hybridization (FISH) [42]. Despite such sparse evidence, a recent case report by Glover et al. (2025) [43] indeed presents the case of a 64-year-old patient with HPV-associated vaginal cancer who received five cycles of EV (d1,8 q3w) and pembrolizumab (d1 q3w) followed by external beam radiation (55 Gy, 20 fractions) combined with weekly cisplatin (20 mg/m^2^); a treatment strategy which evolved after an initial diagnostic conundrum due to pathological features mimicking urothelial carcinoma. In this case, Nectin-4 expression analysis was performed *ex-post*, demonstrating a strong membranous expression of viable tumor cells; next-generation sequencing identified a TMB of 14 mut/MB. Within a follow-up period of 6 months, the patient showed no radiographic or laboratory-based evidence of disease; however, the extent to which EV, pembrolizumab (FDA approved for tumors with TMB > 10 mut/MB [44,45]) or their combination contributed to the favorable clinical outcome cannot be determined [43]. That said, in our study we determined—for the first time to our knowledge—a prevalence of strong Nectin-4 staining in 21% as well as a moderate staining in 24% of PVC, according to the IRS_total_ score. In our cohort, assessment of the Youden Index for Nectin-4 expression demonstrated low discriminatory performance of the evaluated cut-offs (IRS_total_ YI = 0.2 and IRS_memb_ YI = 0.1167), suggesting limited capacity to stratify patients according to survival outcomes. These observations are in line with the non-significant log-rank test results and do not support a prognostic role for Nectin-4 expression with regard to survival within this cohort. Furthermore, we did not observe a significant association with recurrence or metastasis.

Although prospective IHC assessment of Nectin-4 expression was not required for eligibility within the EV-201 phase II trial [46], Klümper et al. distinctly reported the predictive value of Nectin-4 expression (evaluating the membranous protein expression and Nectin-4 amplifications in metastatic urothelial cancer) to targeted therapy [47,48]. This highlights the importance of such precise immunohistochemical analysis, as presented here, prior to establishment of clinical trials in PVC.

### 3.3. Implications for Practice and Future Research

Aiming to individualize patient treatment, tumor-agnostic, biomarker-driven therapeutic strategies represent an innovative approach that overcomes traditional organ- and site-specific treatment paradigms [49]. However, clinical studies often consider tumor entities based on epidemiological frequency and biomarker prevalence [9], which results in a relative lack of evidence for rare tumor entities such as PVC [21]—a gap that the present study aims to partially address.

The high prevalence of HER2 expression observed in this study supports routine HER2 immunohistochemical testing [5]. From a clinical perspective, we advocate for early testing in all patients with locally advanced, recurrent, or metastatic PVC, even in hospital settings without an established institutional molecular tumor board. This approach may enable early identification of positive cases also in peripheral hospitals and facilitates referral to tertiary centers for definitive evaluation and potential tumor-agnostic treatment. Future studies should evaluate the role of FISH-based detection of HER amplification not only in IHC-positive tumors but also in tumors with an IHC score of 1+, as suggested by data in cervical cancer [21]. The high prevalence of Nectin-4 expression in PVC demonstrated in this study generally supports the inclusion of Nectin-4-positive PVC in clinical trials targeting Nectin-4. Furthermore, a combined biomarker analysis conducted within a structured study protocol would facilitate the assessment of optimal IHC interpretation methods (including IRS, H-score, and membranous versus cytoplasmic staining patterns). In addition, future studies should address whether Nectin-4 immunohistochemical results are influenced by different antibody clones [41,47]. That said, despite the observed biomarker expression profiles, further evaluation within prospective clinical studies remains a prerequisite before implementation of tumor-agnostic therapies can be considered in routine clinical management of PVC. Importantly, biomarker expression alone is insufficient to establish therapeutic eligibility, as clinical trial-based treatment selection may additionally require molecular confirmation (e.g., assessment of gene amplification), adequate Eastern Cooperative Oncology Group performance status, and measurable disease according to RECIST 1.1 criteria.

### 3.4. Strengths and Weaknesses

The strengths of this study include (i) a well-defined diagnostic classification of PVC using p16 and p53, (ii) proof-of-concept evidence of HER2 expression, demonstrating a high prevalence in PVC and suggesting potential for broader application of HER2-targeted therapeutic strategies, and (iii) the evaluation of Nectin-4 protein expression, providing a biological rationale for Nectin-4-targeted treatment approaches in this rare malignancy.

A distinct limitation of this study is the absence of more comprehensive molecular approaches (e.g., RNA sequencing) and additional in situ hybridization (ISH) analyses. Although our definition of HER2 positivity followed the eligibility criteria applied in the DESTINY-PanTumor02 trial (see manuscript Section 4.1 for details), it should be acknowledged that this approach differs from established diagnostic and therapeutic HER2 assessment algorithms. In current clinical practice, HER2 IHC 2+ expression is generally regarded as equivocal and requires ISH confirmation [50], while therapeutic decision-making typically relies on HER2 IHC 3+ expression or HER2 IHC 2+ expression with documented ISH gene amplification (ISH positivity). Accordingly, our approach allows for semi-quantitative evaluation of HER2 protein expression but does not provide information on HER2 gene amplification status, which was not assessed within the scope of this exploratory analysis. Furthermore, as HPV testing using nucleic acid-based methods was not performed, the present study did not allow for further evaluation of HPV status or assessment of concordance between molecular HPV detection and p16 immunohistochemical expression. Another limitation is the relatively small sample size. However, the latter reflects the rarity of the disease—the cohort size is comparable to those reported in the existing literature on PVC biomarker studies [14,15]. That said, the corresponding limited statistical power of the study, reflected by wide confidence intervals and an increased risk of type II error, particularly in the survival analyses, should be considered when interpreting the findings.

## 4. Materials and Methods

This study cohort is part of a retrospective research project evaluating tissue-derived biomarkers in PVC, comprising patients diagnosed and treated at the University Medical Center of the Johannes Gutenberg University Mainz (2008–2024). Cases were identified through a query of the hospital’s internal, tumor board-maintained records for diagnosis of vaginal cancer. All patients underwent treatment in accordance with the recommendations of the interdisciplinary tumor board and established clinical guidelines. The inclusion criterion was a priori defined as: available tissue specimens (Biobank at Mainz University Medical Center) from patients with histomorphological confirmation of PVC. As part of routine practice, the diagnostic criteria required the clinical and/or histopathological exclusion of concurrent or recent (within the preceding five years) cervical or vulvar carcinoma. Cases with an incomplete tumor diagnostic workup or a diagnosis of non-epithelial malignancy were excluded.

Forty-two patients were retrieved for subsequent case evaluation and analysis; eight patients had to be excluded (reasons: insufficient tissue available, one patient with vaginal melanoma, one patient with carcinoma of the Bartholin gland). This study was approved by the local ethical committee (Ethics Committee of the Rhineland-Palatinate Medical Association; study identification number 2025-18304), and all data were handled in accordance with the Declaration of Helsinki [51]. Explicit patient consent for this study was waived due to the retrospective nature of this study in full accordance with the ethical approval as well as national law. Clinical data were obtained regarding age, tumor stage (International Federation of Gynecology and Obstetrics (FIGO) staging system), histological type, and type of treatment received. Data on OS, recurrence rates and rates of metastasis were received from internal electronic medical records and the state cancer registry (Rhineland-Palatinate).

### 4.1. Immunohistochemistry and Staining Pattern Analysis

Given the rarity of the tumor, either biopsy specimens or surgical resection samples were included, depending on availability. To optimally account for tumor heterogeneity and spatial distribution of marker expression, whole-slide analysis was performed instead of construction of a tissue microarray (TMA). Immunohistochemical analyses were conducted in accordance with standardized and established staining protocols of the routine laboratory using the Dako CoverStainer (Agilent, Santa Clara, CA, USA) and using primary antibodies against p16 (clone JC8, monoclonal mouse antibody; Dako Omnis, Agilent, Santa Clara, CA, USA), p53 (clone DO-7, monoclonal mouse antibody; Dako Omnis, Agilent, USA), HER2 (HercepTest™ mAb pharmDx, clone DG44, monoclonal rabbit antibody; Dako Omnis, Agilent, Santa Clara, CA, USA), and Nectin-4 (clone EPR15613-68, monoclonal rabbit antibody; Abcam, Cambridge, UK). Sections (2 µm) were subjected to antigen retrieval prior to incubation with the primary antibodies at room temperature; for visualization, the Dako EnVision FLEX + detection system (Agilent, Santa Clara, CA, USA) was employed. Positive and negative controls were included for all proteins analyzed as part of the laboratory routine. Slides were scanned with the Nano Zoomer 2.0HT Scanner (Hamamatsu Photonics, Shizuoka, Japan). Interpretation of digital IHC stainings was performed by a resident pathologist (HW) and a board-certified pathologist (CE), with the evaluators blinded to the clinical data.

For evaluation of the p16^INK4a^ protein, a so-called “abnormal diffuse positive” continuous nuclear and cytoplasmic expression of neoplastic cells was used as a surrogate indicator of HPV-associated neoplasms, in accordance with the *2020 WHO Classification of Female Genital Tumors* [52]. Non-specific or “patchy” p16 staining patterns were regarded as non-contributory. As defined by Tessier-Cloutier et al., p53 staining patterns in SCC were classified as either mutant (basal overexpression, parabasal/diffuse overexpression, cytoplasmic expression, or absence of staining) or wildtype (mid-epithelial or scattered expression) [53]; the mutant staining pattern in adenocarcinomas was defined as overexpression (>80% of cells), absence of staining, or cytoplasmatic staining [54,55]. HER2 expression was scored on a scale from 0 to 3+ in accordance with the current ASCO/College of American Pathologists guidelines for evaluation of gastric cancer (in alignment with the DESTINY-PanTumor02 trial) [9,50,56]. Positivity was defined as HER2 overexpression IHC 3+ or IHC 2+, consistent with the DESTINY-PanTumor02 trial [9]; HER2 expression IHC ≤ 1+ was considered negative. In alignment with an recent approach on the evaluation of Nectin-4 assessment in vulvar cancer, Nectin-4 protein expression was quantified using an immunoreactive score (IRS) based on staining intensity (0 = negative, 1 = weak, 2 = moderate, 3 = strong) multiplied by percentage of positive stained cells (0% = 0, 1–10% = 1, 11–50% = 2, 51–100% = 3), resulting in overall IRS rankings of negative (IRS: 0), weak (IRS: 1–3), moderate (IRS: 4–6), and strong (IRS: 7–9) [41]. As two distinct approaches to staining evaluation exist—namely, the combined assessment of prominent cytoplasmic and membranous staining (IRS_total_) and the exclusive assessment of membranous staining as the pharmaceutical precondition for ADC binding (IRS_memb_)—both scoring methods were reported in this study [42,57].

### 4.2. Statistics

Clinicopathological data, OS, local recurrence, and metastatic risk were descriptively analyzed (GraphPad, Boston, MA, USA; version 11) in relation to the histomorphological biomarkers of interest. Data distribution normality was evaluated using the Shapiro–Wilk test. Intergroup differences in binary variables (positive vs. negative: p16, p53, HER2) were evaluated using Fisher’s exact test (non-normally distributed data) or Welch’s *t*-test (Gaussian distribution). Ordinal variables (IRS: Nectin-4) were compared using the Mann–Whitney test/Kruskal–Wallis test (non-normally distributed data) or one-way ANOVA (Gaussian distribution). Furthermore, the optimal cut-off for Nectin-4 subgrouping was identified through receiver operating characteristic (ROC) curve analysis and subsequent assessment of YI across all potential thresholds. The prognostic impact on OS was assessed for all biomarkers of interest using the log–rank (Mantel–Cox) test. For all analyses, the level of statistical significance was set at α < 0.05.

## 5. Conclusions

This retrospective analysis identifies a notable prevalence of HER2 and Nectin-4 expression in PVC, providing a biological rationale for further exploration of tumor-agnostic therapeutic strategies involving ADCs such as T-Dxd and EV for individualized treatment of vaginal tumors. Given the exploratory nature, single-center design, and limited sample size of this study, these findings should be considered hypothesis-generating and require validation in larger, prospective cohorts before potential clinical translation.

## Figures and Tables

**Table 1:** Clinicopathological characteristics of the PVC study cohort.

Clinicopathological Data	n = 34 (≈100%)
age (median; IQR)	62 (53–73) years
FIGO I	11 (32.35%)
FIGO II	6 (17.65%)
FIGO III	11 (32.35%)
FIGO IVa	2 (5.88%)
FIGO IVb	4 (11.76%)
first-line therapy: surgery	13 (38.24%)
first-line therapy: chemoradiotherapy	12 (35.29%)
first-line therapy: surgery + radiotherapy/chemoradiotherapy	9 (26.47%)
squamous cell carcinoma	30 (88.24%)
adenocarcinoma	4 (11.76%)
local recurrence	14 (41.18%)
metastasis	12 (35.29%)
deceased	10 (29.41%)

**Table 2:** Immunohistochemical expression of p16, p53, HER2, and Nectin-4 in the PVC cohort (n = 34).

Immunohistochemical Staining	n = 34 (≈100%)
p16 positive	27 (79.41%)
p16 negative	7 (20.59%)
p53 mutant pattern	5 (14.71%)
p53 wildtype pattern	29 (85.29%)
HER2 score 0	17 (50%)
HER2 score 1	12 (35.29%)
HER2 score 2	4 (11.76%)
HER2 score 3	1 (2.94%)
Nectin-4 IRS_total_ score: negative	6 (17.65%)
Nectin-4 IRS_total_ score: weak	13 (38.24%)
Nectin-4 IRS_total_ score: moderate	8 (23.53%)
Nectin-4 IRS_total_ score: strong	7 (20.59%)
Nectin-4 IRS_memb_ score: negative	21 (61.76%)
Nectin-4 IRS_memb_ score: weak	7 (20.59%)
Nectin-4 IRS_memb_ score: moderate	6 (17.65%)
Nectin-4 IRS_memb_ score: strong	0

**Table 3:** Comparison of clinicopathological features stratified by HER2 expression status. Younger age, HPV-independent carcinomas as well as adenocarcinomas are significantly associated with HER2 positivity (Fisher’s exact test). ^a^ Welch’s *t*-test, ^b^ Fisher’s exact test. Statistically significant results are shown in bold.

Variable of Interest	HER2 Positive	HER2 Negative	*p*
Age (years)	48 (34.5–60)	63 (55.5–75.5)	**0.0483** ^a^
FIGO stage (I–IVb)	5 (14.71%)	29 (85.29%)	>0.9999 ^b^
Histological type: squamous cell carcinoma	1 (3.33%)	29 (96.67%)	**0.0001** ^b^
Histological type: adenocarcinoma	4 (100%)	0	
p16 positive	1 (3.7%)	26 (96.3%)	**0.0035** ^b^
p16 negative	4 (57.14%)	3 (42.86%)	
Tissue specimen: surgical specimen	3 (15.79%)	16 (84.21%)	>0.9999 ^b^
Tissue specimen: biopsy material	2 (13.33%)	13 (86.67%)	
Development of local recurrence	2 (14.29%)	12 (85.71%)	>0.9999 ^b^
Development of metastasis	1 (8.33%)	11 (91.67%)	0.6347 ^b^

## Data Availability

The data are not publicly available due to privacy and ethical restrictions. The data presented in this study are available on request from the corresponding author.

## Supplementary Materials

The following supporting information can be downloaded at https://www.mdpi.com/article/10.3390/ijms27157018/s1.

## Abbreviations

The following abbreviations are used in this manuscript:

ADC	Antibody-drug conjugates
CAM	Cellular adhesion molecule
CLDN18.2	Claudin-18.2
ESGO	European Society of Gynaecological Oncology
EV	Enfortumab vedotin
FDA	U.S. Food and Drug Administration
FIGO	Federation of Gynecology and Obstetrics
FISH	Fluorescence in situ hybridization
FOLR1	Folate Receptor Alpha
HER2	Human Epidermal Growth Factor Receptor 2
IHC	Immunohistochemistry
ISH	In situ hybridization
IRS	Immunoreactive score
MAPK	Mitogen-activated protein kinase
MSI	Microsatellite instability
NCCN	National Comprehensive Cancer Network
Nectin-4	Poliovirus receptor-like 4
NOS	Not otherwise specified
OS	Overall survival
PD-L1	Programmed death-ligand 1
PI3K/Akt	Phospho-inositide 3-kinase/Akt pathway
PVC	Primary vaginal cancer
SCC	Squamous cell carcinoma
T-Dxd	Trastuzumab deruxtecan
TMA	Tissue microarray
TMB	Tumor mutational burden
TROP2	Trophoblast cell surface antigen 2
