# Supplementary material for: Evaluation of HER2 and Nectin-4 Expression in Primary Vaginal Cancers
Source: Int J Mol Sci. 2026 Aug 5;27(15):7018. doi: 10.3390/ijms27157018 (PMC13467148; doi:10.3390/ijms27157018)
Supplement: Supplementary file 1 [file ijms-27-07018-s001.zip › ijms-4432326-supplementary.pdf]

SUPPLEMENTARY MATERIAL

Title: Evaluation of HER2 and Nectin-4 Expression in Primary Vaginal Cancers

Table S1. p53 staining patterns of squamous cell carcinomas as defined by Tessier-Cloutier et al. (Mod Pathol. 2020 Aug;33(8):1595-1605. DOI: 10.1038/s41379-020-0524-1). Scattered and mid-epithelial (basal sparing) expression is considered p53 wild-type.

**p53 expression pattern**	**n=28 (≈100%)**
1=scattered	24 (71%)
2=mid-epithelial (basal sparing)	2 (6%)
3=basal overexpression	2 (6%)
4=parabasal/diffuse overexpression	2 (6%)
5=absent	0
6=cytoplasmatic	0

Table S2. Comparison of clinicopathological features stratified by p16 status. [a]Welch's t-test, [b]Fisher's exact test.

**variable of interest**	**p16 positive**	**p16 negative**	**p**
Age (years)	62 (54-73)	62 (44-73)	0.4676[a]
FIGO stage (I-IVb)	7 (20.59%)	27 (79.41%)	0.5337[b]
Histological type: squamous cell carcinoma	26 (86.67%)	4 (13.33%)	**0.0211**[b]
Histological type: adenocarcinoma	1 (25%)	3 (75%)	
Development of local recurrence	10 (71.43%)	4 (28.57%)	0.4099[b]
Development of metastasis	10 (83.33%)	2 (16,67%)	>0.9999[b]

Table S3. Comparison of clinicopathological features stratified by p53 staining pattern. [a]Welch's t-test, [b]Fisher's exact test.

**variable of interest**	**p53 mutant pattern**	**p53 wildtype pattern**	**p**
Age (years)	63 (57-77)	62 (49-73)	0.4639[a]
FIGO stage (I-IVb)	5 (14.71%)	29 (85.29%)	0.2807[b]
Histological type: squamous cell carcinoma	4 (13.33%)	26 (86.67%)	0.4879[b]
Histological type: adenocarcinoma	1 (25%)	3 (75%)	
Development of local recurrence	3 (21.43%)	11 (78.57%)	0.6272[b]
Development of metastasis	1 (8.33%)	11 (91.67%)	0.6347[b]

Supp. Table S4. Comparison of clinicopathological features stratified by Nectin-4 IRS_{total} and Nectin-4 IRS_{memb} status. [a]Ordinary one-way ANOVA, [b]Kruskal-Wallis test, [c]Mann-Whitney test.

**variable of interest**	**Nectin-4 IRS_{total}: negative**	**Nectin-4 IRS_{total}: weak**	**Nectin-4 IRS_{total}: moderate**	**Nectin-4 IRS_{total}: strong**	**p**
age (years)	62 (57.25-75)	62 (45.4-73)	61.5 (48.5-79.5)	62 (61-63)	0.8999[a]
FIGO stage (I-IVb)	6 (17.65%)	13 (38.24%)	8 (23.53%)	7 (20.59%)	0.1386[b]
histological type: squamous cell carcinoma	5 (16.67%)	10 (33.33%)	8 (23.53%)	7 (20.59%)	0.1595[c]
histological type: adenocarcinoma	1 (25%)	3 (75%)	0	0	
p16 positive	3 (11.11%)	10 (37.04%)	8 (29.63%)	6 (22.22%)	0.0623[c]
p16 negative	3 (42.86%)	3 (42.86%)	0	1 (14.29%)	
Tissue: surgical specimen	5 (26.32%)	7 (36.84%)	4 (21.05%)	3 (15.79%)	0.1999[c]
Tissue: biopsy material	1 (6.7%)	6 (40%)	4 (26.67%)	4 (26.67%)	
Development of local recurrence	4 (28.57%)	5 (35.71%)	4 (28.57%)	1 (7.14%)	0.1509[c]
Development of metastasis	2 (16.67%)	5 (41.67%)	3 (25%)	2 (16.67%)	0.8407[c]
**variable of interest**	**Nectin-4 IRS_{memb}: negative**	**Nectin-4 IRS_{memb}: weak**	**Nectin-4 IRS_{memb}: moderate**	**Nectin-4 IRS_{memb}: strong**	**p**
age (years)	61 (49-71)	61 (46-78)	62.5 (60-81.5)	-	0.5898[a]
FIGO stage (I-IVb)	21 (61.76%)	7 (20.59%)	6 (17.65%)	0	0.3373[b]
histological type: squamous cell carcinoma	18 (60%)	6 (20%)	6 (20%)	0	0.5427[c]
histological type: adenocarcinoma	3 (75%)	1 (25%)	0	0	
p16 positive	16 (59.26%)	6 (22.22%)	5 (18.52%)	0	0.6387[c]
p16 negative	5 (71.43%)	1 (14.29%)	1 (14.29%)	0	
Tissue: surgical specimen	13 (68.42%)	4 (21.05%)	2 (10.53%)	0	0.3418[c]
Tissue: biopsy material	8 (53.33%)	3 (20%)	4 (26.67%)	0	
Development of local recurrence	10 (71.43%)	3 (21.43%)	1 (7.14%)	0	0.2698[c]
Development of metastasis	9 (75%)	1 (8.33%)	2 (16.67%)	0	0.3872[c]

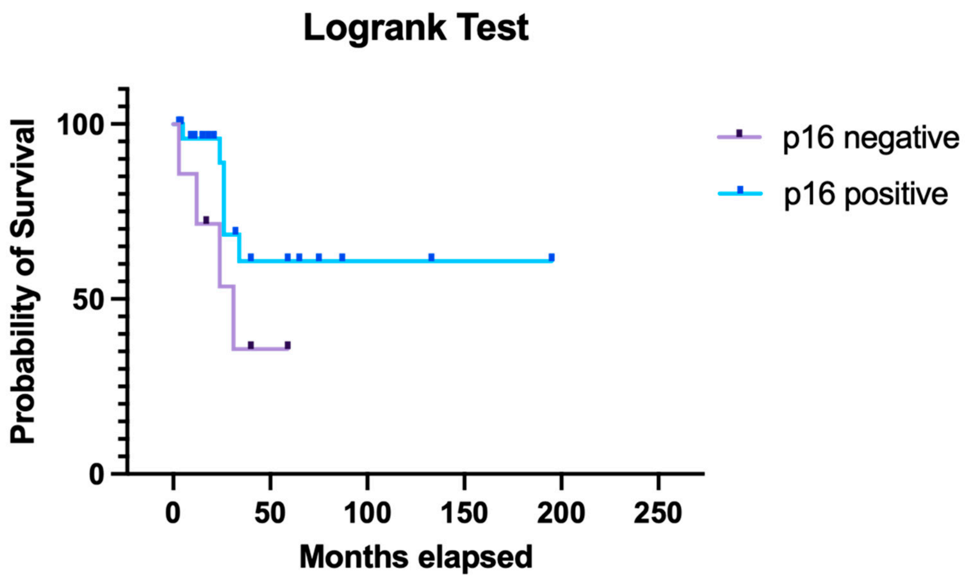

Figure S1. Kaplan–Meier curve illustrating the impact of p16 expression status on overall survival; the p-value was calculated using the log-rank (Mantel–Cox) test.
